# Assessment of the Quality Management System for Clinical Nutrition in Jiangsu: Survey Study

**DOI:** 10.2196/27285

**Published:** 2021-09-27

**Authors:** Jin Wang, Chen Pan, Xianghua Ma

**Affiliations:** 1 First Affiliated Hospital of Nanjing Medical University Nanjing China

**Keywords:** quality management system, human resource management, artificial intelligence, online health, health science, clinical nutrition, online platform, health platform, nutrition, patient education, dietitian

## Abstract

**Background:**

An electronic system that automatically collects medical information can realize timely monitoring of patient health and improve the effectiveness and accuracy of medical treatment. To our knowledge, the application of artificial intelligence (AI) in medical service quality assessment has been minimally evaluated, especially for clinical nutrition departments in China. From the perspective of medical ethics, patient safety comes before any other factors within health science, and this responsibility belongs to the quality management system (QMS) within medical institutions.

**Objective:**

This study aims to evaluate the QMS for clinical nutrition in Jiangsu, monitor its performance in quality assessment and human resource management from a nutrition aspect, and investigate the application and development of AI in medical quality control.

**Methods:**

The participants for this study were the staff of 70 clinical nutrition departments of the tertiary hospitals in Jiangsu Province, China. These departments are all members of the Quality Management System of Clinical Nutrition in Jiangsu (QMSNJ). An online survey was conducted on all 341 employees within all clinical nutrition departments based on the staff information from the surveyed medical institutions. The questionnaire contains five sections, and the data analysis and AI evaluation were focused on human resource information.

**Results:**

A total of 330 questionnaires were collected, with a response rate of 96.77% (330/341). A QMS for clinical nutrition was built for clinical nutrition departments in Jiangsu and achieved its target of human resource improvements, especially among dietitians. The growing number of participating departments (an increase of 42.8% from 2018 to 2020) and the significant growth of dietitians (*t*_93.4_=–0.42; *P*=.02) both show the advancements of the QMSNJ.

**Conclusions:**

As the first innovation of an online platform for quality management in Jiangsu, the Jiangsu Province Clinical Nutrition Management Platform was successfully implemented as a QMS for this study. This multidimensional electronic system can help the QMSNJ and clinical nutrition departments achieve quality assessment from various aspects so as to realize the continuous improvement of clinical nutrition. The use of an online platform and AI technology for quality assessment is worth recommending and promoting in the future.

## Introduction

From the perspective of medical ethics, patient safety is the core before any other factors within health science. In the application of health science, medical services are inseparable from the safety of patients’ lives and medical ethics. The scope of its practice is composed of statutory and individual components, and includes codes of ethics (eg, health institution, department director, or other national organizations) and other resources [1]. Aiming to protect the patient’s safety while providing accurate medical services, a quality management (QM) system was established at national, provincial, and municipal levels within each single clinical discipline. As the QM Center of Clinical Nutrition in Jiangsu (QMCNJ), it is the system’s responsibility to standardize and improve the professional performance within the province’s tertiary hospitals. Their quality assessment comes from the reported data of all clinical nutrition (CN) departments among these hospitals, such as human and material resources, professional practice, food service operation, patient’s satisfaction, and nutrition education presentations for the hospital and community [1,2]. All of the required information is scheduled based on the Revised 2016 Standards of Construction and Management of Clinical Nutrition Department in Jiangsu, reflects advances in CN department practices during the past 6 years, and replaces the 2010 Standards [3]. Based on the statistical analysis of the outpatients and inpatients with nutrition intervention, the actual medical work’s progress is clarified in the nutrition department of each hospital. Based on the information from food service operations, its satisfaction of nutrition therapy is illustrated as well. All of these data are required to be statistically analyzed by QMCNJ per quarter to provide reliable and credible feedback to each CN department. With these quality assessments, all CN departments can more accurately grasp their own medical quality, define the orientation of its development within the QM system, and find its direction for continuous improvement in the future.

Within this information era, most of the current information and data in health care is network based [4]. An electronic system that automatically collects medical information can provide timely monitoring of patient health and improve the effectiveness and accuracy of medical treatment. From a medical quality perspective, intelligent management systems can improve data curation, reduce human resource costs, and contribute to facilitating continuous improvement. As one of the inventions in the information era, artificial intelligence (AI) has shown its strong adaptability to the network-based health care system. It can be introduced into clinical behavior detection accurately and automatically, and is important for reducing the incidence of treatment errors and ensuring patient safety. However, the amount of digital data has increased substantially after using the online system [5]. A consequence is that data management has become more complex, which has increased the necessity for methods that are able to deal with the quality assessment of digital information. From the perspective of QM and the disciplined development of a medical specialty such as CN, the specific indicators and the evaluation of AI application are important for achieving quality control goals.

To our knowledge, the application of AI into medical service quality assessment has only been minimally evaluated, especially for CN departments. There was a unified platform for all QM centers of various medical specialties set up by the Jiangsu Provincial Health Commission. After its shutdown in October 2017, the QMCNJ became the first center to independently develop and promote the application of its online platform named Jiangsu Province Clinical Nutrition Management Platform (JPCNMP) [6]. This platform does not require any software installation; simply logging on to the website allows an individual to start data reporting, which makes related information collection more convenient. It was officially launched in the Quality Management System of Clinical Nutrition in Jiangsu (QMSNJ) in 2019 and successfully promoted to 70 CN departments within the quality control system. They are required to fill in relevant information regularly in accordance with the regulations of Strengthening the Management of Provincial Medical Quality Management System [7] formulated by the Jiangsu Provincial Health Commission, which was revised in September 2020. Since the platform has been stable for 2 years, its effectiveness in QM remains to be validated. At the same time, the application value of AI and the development of the CN departments in Jiangsu also need to be clarified.

## Methods

### Participants

An online questionnaire was designed by the QMCNJ for employees in 70 CN departments of the tertiary hospitals in Jiangsu Province, which are all members of the QMSNJ. There were 341 staff in total based on the human resource information from the surveyed medical institutions. The questionnaire contained five aspects: hospital information, personal profile, education in QM, scientific research achievements, and nutrition education presentation through the internet. The detailed questions are shown in Multimedia Appendix 1.

### Survey Recruitment and Data Collection

Before distribution, the survey and methods were approved by the QMCNJ and Jiangsu Provincial Health Commission. The data were collected through an online survey (ID: 97003762) through the open-access platform Wen Juan Xing [8]. One code was requested to fill in the questionnaire. An electronic reminder was sent by QMCNJ to the secretary of these 70 CN departments through SMS text messaging, email, JPCNMP, and WeChat. The deadline for completing this survey was November 14, 2020.

### Ethics

Institutional ethics approval was obtained for this study from the Ethics Committee of the First Affiliated Hospital with Nanjing Medical University. This survey presents no risks to the participants nor did it involve any therapeutic intervention. All the personal information within the questionnaire was designed to verify the authenticity of the feedback. After screening valid questionnaires, all personal information was not included in the statistical analysis. As a result, there was no risk of additional use or disclosure of private information. Key informants were assured that confidentiality would be maintained and that findings would be presented in an anonymous fashion.

### Data Analysis

Because information about the CN departments’ human resource and service requirements of related hospitals could be automatically captured through the JPCNMP and the hospital information system (HIS), the risk of error caused by manual filling was avoided. Therefore, these two pieces of information are the most easily obtained and most accurate data for CN departments’ status assessment. In addition, it is the foundation for this research. All of this information was gathered on November 14, 2020. This original data is accessible from the QMCNJ [9].

Only completed questionnaires were included in the data analysis. An overall description was performed to summarize the demographic and professional characteristics of the valid study sample. The current construction of the CN departments and their human resources has been clarified with this description. Comparing it with the corresponding data when the online management system was not activated, which is documented in the survey Current Status of the Clinical Nutrition Departments Among Tertiary Hospitals in Jiangsu Province [10] conducted by our research team in 2018, a preliminary evaluation of the quality control progress was obtained.

The in-depth quality assessment from each participant in the QMSNJ was gathered. Because the JPCNMP was maintained in the trail operation stage in 2018, it has not been uniformly applied in the entire QMSNJ. Therefore, a natural time grouping was formed according to the dates when the two surveys were completed. In 2018, the 49 CN departments that did not use AI tools for QM were the control group, and the corresponding CN departments that were using AI tools for management in 2020 were the observation group. The list of QMSNJ in 2018 was used to filter valid information. Some new departments applied to participate in the quality control system within this period, and their information in 2018 was not surveyed, so they cannot be compared. Another criterion was the consistency between the *numbers of colleges in your department* in the Hospital Information section and the number of documented staff of the CN department in their HIS system. This was used to verify the authenticity of the feedback. The questionnaire was considered valid when these two data matched. A paired sample *t* test was conducted to determine differences between their situation before and after the application of JPCNMP.

### Statistical Method

SPSS Statistics 22.0 (IBM Corp) was used to describe the results; statistical significance was set at *P*≤.05, as shown in Multimedia Appendix 1.

## Results

### Proportion of Valid Feedback

Based on the staff information from the surveyed medical institutions’ HIS, there are 341 employees within all CN departments. On November 1, 2020, once the survey was delivered to each participant, they were given 14 days to complete the survey. All of the collected questionnaires were screened according to the rubrics of valid conditions. With a response rate of 96.77%, a total of 330 valid questionnaires were counted.

### Overall Description of the QCSNJ

There were 70 departments in this QM system in 2020, which increased 42.8% since 2018 (49 departments). In terms of human resources, the total number of employees in all CN departments has increased from 313 to 341 (8.95%) as shown in Table 1.

**Table 1:** Staff of clinical nutrition departments in 2018 and 2020.

	Staff in 2018 (n=313), n (%)	Staff in 2020 (n=346), n (%)
Dietitians	113 (36.1)	104 (30.1)
Clinicians	135 (43.1)	158 (45.7)
Nurses	65 (20.8)	84 (24.3)

### Human Resources in CN Departments

Of the 330 valid responses, most were clinicians (n=158, 46.33%), followed by dietitians (n=104, 30.49%) and nurses (n=84, 24.63%), as shown in Table 1. As the registered dietitian/registered dietitian nutritionist (RD/RDN) was only certificated by the Chinese Nutrition Society after 2016 and is still not an employment requirement for professionals in CN departments, the number of RD/RDNs from this survey was only 92 (26.98%). Among this population, 45 clinicians, 35 dietitians, and 12 nurses have earned the RD/RDN certification.

### Progress of Management in CN Departments

There are 49 departments that participated in the 2018 survey, which provided the basis of this comparison. As shown in Multimedia Appendix 2, 48 CN departments were included in this part of the analysis (1 CN department was excluded as they provided different staff numbers than their hospital’s HIS). The only significant increase was observed in the number of dietitians within 2 years, while the other changes were not apparent at the same time.

Considering the changes in general medical requirements within these 2 years, which could be observed through the data of hospital beds in HISs, we chose the ratio of professionals in CN departments to hospital beds as another evaluation index. As shown in Multimedia Appendix 3, this ratio belongs to one of the criteria issued by the Jiangsu Provincial Health Commission for hospital accreditation. Similar to the insignificant resource development in CN departments, the ratio of various professionals to hospital beds (total staff, clinician, dietitian) was not noticeable. Only the ratio between nurse and hospital beds indicated a significant decrease within these 2 years.

## Discussion

### Principal Findings

The significant growth in human resources and the number of CN departments involved in the QM system in Jiangsu show promising development of professionals within the nutrition area. After showing the staff shortage in 2018, the QMCNJ focused on improving all quality control system departments. This progress shows that the importance of CN medicine in health care services had been a focus for health institutions, department managers, and other national organizations such as the Jiangsu Provincial Health Commission [11]. The ratio criteria between CN department professionals to hospital beds issued by the Jiangsu Provincial Health Commission for hospital accreditation is 1:200 (0.50 × 10^–2^). Compared with the mean value from this research (0.36 × 10^–2^), the CN departments are still in need of human resources and should make more effort toward resource management.

The increasing number of departments that participated in the QMSNJ brought their staff into the QM system of CN, which led to an increased total amount of human resources. However, most of this growth resulted from clinicians and nurses instead of dietitians. The dietitians in Jiangsu decreased from 113 to 104, while the clinicians increased by 13 and nurses increased by 29. This led to a decrease in the professionals who had graduated from nutrition majors and desired to provide nutrition-related medical services. Based on the human resource profile from surveyed hospitals and the documentation in the JPCNMP, most of the clinicians employed in CN departments were educated as physicians such as gastroenterologists or endocrinologists. As a result, although the hospital, the human resource department, and the CN department director realized the importance of CN medicine and desired to improve its staff resource, the CN departments still lacked certified dietitians. This vagueness may be induced by the late development of CN in China. The certification of RD/RDNs was officially organized by the Chinese Nutrition Society in the past 5 years. Compared to the lack of RD/RDNs throughout the province 2 years before, there were 92 (26.98%) now accounted for, and 38.04% were from nutrition majors. Although it is still less ideal than the proportion of RD/RDN in US CN medicine (98.6%) [12], breakthroughs were achieved within this time period.

The advancement of CN departments’ staff resources might be mainly related to the expansion of the QMSNJ, which has proved the QMCNJ has achieved initial results in the QM of human resources engaged in CN in Jiangsu in recent years. However, judging from the comparison of the personnel situation of the 48 original CN departments, which had been involved in the 2018 QMSNJ and participated in the last construction survey [10], the number of their dietitians has increased significantly (*t*_93.4_=–0.42; *P*=.02). Simultaneously, the number of clinicians in relevant departments is the same, and the number of nurses has shown a decreasing trend without statistical significance. From the perspective of balancing the CN resources and health service requirements, which could be illustrated by the ratio of professionals in CN departments to hospital beds, the decrease was only observed in nurse numbers (*t*_85.5_=0.19; *P*=.05). At the same time, no obvious change has been found in the number of clinicians nor dietitians. These results confirmed that the medical institution and the department’s director had realized the importance of a solid foundation of professional staff to improve the quality of specialist work and ensure patient safety. These 48 CN departments have continuously increased their emphasis on CN medicine, and the QMCNJ has promoted RD/RDN resources within clinical trials by improving CN department’s professionalism. Within the *Education in QM* part of this questionnaire, 327 of 330 employees took part in the QM training held through the online platform JPCNMP by the QMCNJ in 2020. In the exam after the course, the passing rate of trainees was 99.20%.

The expansion of the organization of the QMCNJ has been proved through this research and the rising focus on CN medicine and human resource management by medical institutions and health commissions. All these signs of progress are inseparable from the introduction of AI instruments. With the implementation of the specialized online platform JPCNMP after 2019, a real-time quality assessment of clinic nutrition departments’ daily work could be observed by themselves and by the QMCNJ. The platform functions involve quality assessment, information communication, personnel education, training, etc. Through the log-in interface of the CN department, the department director and the secretary can browse its historical QM data and grasp the current status and trends of its medical service quality and department management quality. Relevant problems will be exposed, and adjustments can be implemented in time. Through the log-in interface of QMCNJ, the center specialists can browse each department’s data within the QMSNJ. The JPCNMP system has installed automatic warnings for departments and quality control centers of abnormal information such as inconsistencies, missing values, outliers, and similarities. It provides a great advantage for the QMCNJ to adjust its QM methods based on the current status of CN departments and quality assessment throughout Jiangsu, and to apply management policies to the Jiangsu Provincial Health Commission.

Apart from data collection and quality assessment, the JPCNMP is convenient for the QMCNJ to publish the latest QM guidelines, the QM progress of CN departments in Jiangsu, the frontier QM research trends, etc. The application of this AI technology is effectively saving the resource consumption caused by traditional forms such as filling out information on paper and on-site supervision. Consistent with the trend of the information technology revolution of our era, AI provides an automated method and various rules that are able to deal with the quality assessment of big data for health care [4]. Its advantages have been reported in multiple disciplines such as geothermal systems [12], medical centers [4], clinical laboratories [13], and medical QM [14]. The JPCNMP provides a freely available, open-source tool in data collection for CN departments of the QMSNJ, which achieves the goal of intelligent management. The AI still needs the QMCNJ to achieve data curation and evaluation. As a result, the QMCNJ highlights the importance of the QM data assessment by developing and continuously revising the index evaluation standards since its foundation in 2010. Similar to any data analysis service, the most crucial process of the JPCNMP is the data quality assessment, which is related to the evaluation of data metrics, the organizational structure of the data, and the overall information management [4,15]. Rather than the initial QM system requiring artificial analysis of quality control data, it will be more accurate and reliable if an automated framework effectively manages the quality of the JPCNMP’s data in the future.

### Suggestions

We offer three suggestions for future development from this study. First, the Standards of Construction and Management of Clinical Nutrition Department in Jiangsu must be revised to reflect advances in CNM practice during the past 5 years and replace the 2016 revision. A specific standard of professional performance for RD/RDNs should be included to guarantee the quality of human resources in nutrition fields. Second, it is essential to provide lifelong learning and professional development opportunities for RD/RDNs. The five levels of proficiency [1] (novice, advanced beginner, competent, proficient, and expert) during the acquisition and development of knowledge and skills should be introduced to the QMSNJ. It not only attracts high-quality talent, encourages professional development, and achieves individual professional goals but also optimizes the quality of human resources of CN departments at the same time. Third, the automated information capture has been clarified to be suitable for the medical internet-based quality control. The AI technology of the JPCNMP should keep improving its intelligence by alliances with other management systems, such as HIS workflow or other advanced human resource management systems. Therefore, the professional development of employees could be suggested by both the QM organization and the medical institution.

## Appendix

Multimedia Appendix 1 Online survey for members of the Quality Management System of Clinical Nutrition in Jiangsu in 2020.

Multimedia Appendix 2 Comparison of staffs in clinical nutrition departments between 2018 and 2020.

Multimedia Appendix 3 Ratios between professionals to hospital beds between 2018 and 2020.

## Abbreviations

AI: artificial intelligence
CN: clinical nutrition
HIS: hospital information system
JPCNMP: Jiangsu Province Clinical Nutrition Management Platform
QM: quality management
QMCNJ: Quality Management Center of Clinical Nutrition in Jiangsu
QMS: quality management system
QMSNJ: Quality Management System of Clinical Nutrition in Jiangsu
RD/RDN: registered dietitian/registered dietitian nutritionist
