# Supplementary material for: Assessment of the Quality Management System for Clinical Nutrition in Jiangsu: Survey Study
Source: JMIR Form Res. 2021 Sep 27;5(9):e27285. doi: 10.2196/27285 (PMC8506260; doi:10.2196/27285)
Supplement: Multimedia Appendix 1 [file formative_v5i9e27285_app1.docx]

Table 1 Online Survey for Members of QMSNJ in 2020

| **Online Survey for members of QMSNJ in 2020** | |
| --- | --- |
| **Hospital Information** | Name of your Medical Institution |
|  | The Category of your Institution |
|  | The Specialty Category of your Clinical Nutrition Department (CND) |
|  | Numbers of Colleges in your Department |
| **Personal Profile** | Your Name |
|  | Your Gender |
|  | Your Position |
|  | Your Title |
|  | Are you a certified physician? |
|  | Are you a registered dietitian (RD)? |
|  | Are you a certified nurse? |
| **Develop and Expand Knowledge in QM** | Did you notice the 2020 QMSNJ Training? |
|  | Did you participated in the 2020 QMSNJ Training? |
|  | Date of the 2020 QMSNJ Training |
|  | Content of the 2020 QMSNJ Training |
| **Scientific Research Achievements**  **(from 2018 to 2020)** | List of your current researches |
|  | List of your published research papers |
|  | List of your published monographs |
|  | List of your certified copyrights |
| **Medical Service though Internet**  **(from 2018 to 2020)** | Whether you have taken part in your department`s online medical service? |
|  | The website of your department`s online medical service |
|  | The frequency of your department`s online medical service |
|  | Numbers of the patients in your department`s online medical service |
|  | Whether you have carried out online medical service on your own? |
|  | The website of your own online medical service |
|  | The frequency of your own online medical service |
|  | Numbers of the patients in your own online medical service |
|  | Have you participated in any nutrition education presentation online? |
|  | Numbers of your online nutrition education presentation |
|  | Your online nutrition education video URL |
