# Supplementary material for: Assessment of the Quality Management System for Clinical Nutrition in Jiangsu: Survey Study
Source: JMIR Form Res. 2021 Sep 27;5(9):e27285. doi: 10.2196/27285 (PMC8506260; doi:10.2196/27285)
Supplement: Multimedia Appendix 2 [file formative_v5i9e27285_app2.docx]

Table 2. Comparation of staffs in CND between 2018 and 2020 (Paired sample t-test)

|  | **Year** | **Mean** | **Standard Deviation** | **P** | **T** |
| --- | --- | --- | --- | --- | --- |
| **Total Staff** | 2018 | 6.50 | 4.02 | 0.50 | 0.21 |
|  | 2020 | 6.29 | 2.80 |  |  |
| **Total Clinician** | 2018 | 2.81 | 2.13 | 0.50 | -0.10 |
|  | 2020 | 2.92 | 1.78 |  |  |
| **Total Dietitian** | 2018 | 1.90 | 1.54 | 0.02 | -0.42 |
|  | 2020 | 2.31 | 1.65 |  |  |
| **Total Nurse** | 2018 | 1.33 | 1.11 | 0.09 | 0.25 |
|  | 2020 | 1.08 | 1.09 |  |  |
