# Supplementary material for: Assessment of the Quality Management System for Clinical Nutrition in Jiangsu: Survey Study
Source: JMIR Form Res. 2021 Sep 27;5(9):e27285. doi: 10.2196/27285 (PMC8506260; doi:10.2196/27285)
Supplement: Multimedia Appendix 3 [file formative_v5i9e27285_app3.docx]

Table 3. Ratios between professionals to hospital beds between 2018 and 2020

|  | **Year** | **Mean%** | **Standard Deviation%** | **P** | **T** |
| --- | --- | --- | --- | --- | --- |
| **Total Staff** | 2018 | 0.40 | 0.20 | 0.07 | 0.45 |
|  | 2020 | 0.36 | 0.18 |  |  |
| **Clinician** | 2018 | 0.18 | 0.10 | 0.48 | 0.08 |
|  | 2020 | 0.17 | 0.10 |  |  |
| **Dietitian** | 2018 | 0.12 | 0.09 | 0.82 | -0.03 |
|  | 2020 | 0.12 | 0.09 |  |  |
| **Nurse** | 2018 | 0.09 | 0.09 | 0.05 | 0.19 |
|  | 2020 | 0.07 | 0.08 |  |  |
